# Supplementary material for: A UMLS-based spell checker for natural language processing in vaccine safety
Source: BMC Med Inform Decis Mak. 2007 Feb 12;7:3. doi: 10.1186/1472-6947-7-3 (PMC1805499; doi:10.1186/1472-6947-7-3)
Supplement: Additional file 13 — RAP application directory. Sets up directory for RAP (RDF application for PHP) source code [file 1472-6947-7-3-S13.gz › rap/doc/phpdoc/__filesource/fsource_model__modelModelFactory.php.html]

xml version="1.0" encoding="iso-8859-1"?


File Source for ModelFactory.php


# Source for file ModelFactory.php

Documentation is available at ModelFactory.php

```
1. <?php
2. /**
3. * ----------------------------------------------------------------------------------
4. * Class: ModelFactory
5. * ----------------------------------------------------------------------------------
6. *
7. * @package     model
8. */


11. /**
12. * ModelFactory is a static class which provides methods for creating different
13. * types of RAP models. RAP models have to be created trough a ModelFactory
14. * instead of creating them directly with the 'new' operator because of RAP's
15. * dynamic code inclusion mechanism.
16. *
17. * <BR><BR>History:
18. * <LI>10-05-2004                : First version of this class.</LI>
19. *
20. * @version  V0.9.1
21. * @author Daniel Westphal <mail at d-westphal.de>
22. *
23. *
24. * @package     model
25. * @access    public
26. ***/
27. class ModelFactory
28. {
29. /**
30. * Returns a MemModel.
31. * You can supply a base URI
32. *
33. * @param   string  $baseURI
34. * @return    object    MemModel
35. * @access    public
36. */
37. function & getDefaultModel($baseURI = null)
38. {
39. return ModelFactory::getMemModel($baseURI);
40. }

42. /**
43. * Returns a NamedGraphSetMem.
44. * You can supply a GraphSet name.
45. *
46. * @param string $graphSetId
47. * @param string $uri
48. * @access    public
49. */
50. function & getDatasetMem($graphSetId = null)
51. {
52. include(RDFAPI_INCLUDE_DIR . PACKAGE_DATASET);
53. return new DatasetMem($graphSetId);
54. }

56. /**
57. * Returns a MemModel.
58. * You can supply a base URI
59. *
60. * @param   string  $baseURI
61. * @return    object    MemModel
62. * @access    public
63. */
64. function & getMemModel($baseURI = null)
65. {
66. return new MemModel($baseURI);
67. }

69. /**
70. * Returns a DbModel with the database connection
71. * defined in constants.php.
72. * You can supply a base URI. If a model with the given base
73. * URI exists in the DbStore, it'll be opened.
74. * If not, a new model will be created.
75. *
76. * @param   string  $baseURI
77. * @return    object    DbModel
78. * @access    public
79. */
80. function & getDefaultDbModel($baseURI = null)
81. {
82. $dbStore = ModelFactory::getDbStore();
83. return ModelFactory::getDbModel($dbStore,$baseURI);
84. }

86. /**
87. * Returns a new DbModel using the database connection
88. * supplied by $dbStore.
89. * You can supply a base URI. If a model with the given base
90. * URI exists in the DbStore, it'll be opened.
91. * If not, a new model will be created.
92. *
93. * @param   object    DbStore  $dbStore
94. * @param   string  $baseURI
95. * @return    object    DbModel
96. * @access    public
97. */
98. function & getDbModel($dbStore, $baseURI = null)
99. {
100. if ($dbStore->modelExists($baseURI))
101. return $dbStore->getModel($baseURI);

103. return $dbStore->getNewModel($baseURI);
104. }

106. /**
107. * Returns a database connection with the given parameters.
108. * Paramters, which are not defined are taken from the constants.php
109. *
110. * @param   string   $dbDriver
111. * @param   string   $host
112. * @param   string   $dbName
113. * @param   string   $user
114. * @param   string   $password
115. * @return    object    DbStore
116. * @access    public
117. */
118. function & getDbStore($dbDriver=ADODB_DB_DRIVER, $host=ADODB_DB_HOST, $dbName=ADODB_DB_NAME,
119. $user=ADODB_DB_USER, $password=ADODB_DB_PASSWORD)
120. {
121. return new DbStore($dbDriver, $host, $dbName,$user, $password);
122. }

124. /**
125. * Returns a InfModelF.
126. * (MemModel with forward chaining inference engine)
127. * Configurations can be done in constants.php
128. * You can supply a base URI
129. *
130. * @param   string  $baseURI
131. * @return    object    MemModel
132. * @access    public
133. */
134. function & getInfModelF($baseURI = null)
135. {
136. require_once( RDFAPI_INCLUDE_DIR . PACKAGE_INFMODEL);
137. return new InfModelF($baseURI);
138. }

140. /**
141. * Returns a InfModelB.
142. * (MemModel with backward chaining inference engine)
143. * Configurations can be done in constants.php
144. * You can supply a base URI
145. *
146. * @param   string  $baseURI
147. * @return    object    MemModel
148. * @access    public
149. */
150. function & getInfModelB($baseURI = null)
151. {
152. require_once( RDFAPI_INCLUDE_DIR . PACKAGE_INFMODEL);
153. return new InfModelB($baseURI);
154. }

156. /**
157. * Returns a ResModel.
158. * $modelType has to be one of the following constants:
159. * MEMMODEL,DBMODEL,INFMODELF,INFMODELB to create a resmodel with a new
160. * model from defined type.
161. * You can supply a base URI
162. *
163. * @param   constant  $modelType
164. * @param   string  $baseURI
165. * @return    object    MemModel
166. * @access    public
167. */
168. function & getResModel($modelType, $baseURI = null)
169. {
170. require_once( RDFAPI_INCLUDE_DIR . PACKAGE_RESMODEL);

172. switch ($modelType) {
173. case DBMODEL:
174. $model = ModelFactory::getDbModel($baseURI);
175. break;

177. case INFMODELF:
178. $model = ModelFactory::getInfModelF($baseURI);
179. break;

181. case INFMODELB:
182. $model = ModelFactory::getInfModelB($baseURI);
183. break;

185. default:
186. $model = ModelFactory::getMemModel($baseURI);
187. break;
188. }

190. return new ResModel($model);
191. }

193. /**
194. * Returns an OntModel.
195. * $modelType has to be one of the following constants:
196. * MEMMODEL, DBMODEL, INFMODELF, INFMODELB to create a OntModel
197. * with a new model from defined type.
198. * $vocabulary defines the ontology language. Currently only
199. * RDFS_VOCABULARY is supported. You can supply a model base URI.
200. *
201. * @param       constant      $modelType
202. * @param       string      $baseURI
203. * @return    object        MemModel
204. * @access    public
205. */
206. function & getOntModel($modelType,$vocabulary, $baseURI = null)
207. {
208. require_once( RDFAPI_INCLUDE_DIR . PACKAGE_ONTMODEL);

210. switch ($modelType)
211. {
212. case DBMODEL:
213. $model = ModelFactory::getDBModel($baseURI);
214. break;

216. case INFMODELF:
217. $model = ModelFactory::getInfModelF($baseURI);
218. break;

220. case INFMODELB:
221. $model = ModelFactory::getInfModelB($baseURI);
222. break;

224. default:
225. $model = ModelFactory::getMemModel($baseURI);;
226. }

228. switch ($vocabulary)
229. {
230. case RDFS_VOCABULARY:
231. require_once(RDFAPI_INCLUDE_DIR.'ontModel/'.RDFS_VOCABULARY);
232. $vocab = new RdfsVocabulary();
233. break;

235. default:
236. $vocab = new RdfsVocabulary();
237. break;
238. }

240. return new OntModel($model,$vocab);
241. }
242. }
243. ?>
```

Documentation generated on Thu, 7 Jul 2005 13:42:06 +0200 by phpDocumentor 1.3.0RC3
